# Tuberculosis among transhumant pastoralist and settled communities of south-eastern Mauritania

**DOI:** 10.3402/gha.v9.30334

**Published:** 2016-05-10

**Authors:** Aissata Lô, Anta Tall-Dia, Bassirou Bonfoh, Esther Schelling

**Affiliations:** 1 Institut de Santé et Développement, University Cheikh Anta Diop, Dakar, Senegal; 2 National Institute of Public Health Research, Nouakchott, Mauritania; 3 Centre Suisse de Recherches Scientifiques en Côte d'Ivoire, Abidjan, Côte d'Ivoire; 4 Department of Public Health and Epidemiology, Swiss Tropical and Public Health Institute, Basel, Switzerland; 5 Swiss TPH and the University of Basel, Basel, Switzerland

**Keywords:** tuberculosis, Mauritania, pastoralists, presumptive TB cases, diagnostic capacity

## Abstract

**Background:**

Transhumant pastoralists of Mauritania were assumed to have a high prevalence of tuberculosis (TB) because of reduced access to diagnostic testing. No population-based survey on TB has been published for Mauritania.

**Objective:**

The goal of this study was to estimate the prevalence of presumptive TB cases among mobile pastoralists and villagers in a remote zone of Mauritania.

**Design:**

In the south-eastern province of Hodh Ech Chargui, 250 adult pastoralists and 250 villagers were randomly enrolled using multistage cluster sampling in February 2012. A TB centre nurse examined participants using a standard clinical protocol, and a participant questionnaire was completed. Focus group discussions and interviews were conducted with community members and health personnel, respectively.

**Results:**

Fourteen new presumptive TB cases were identified, leading to an overall prevalence of 2.8%, (95% confidence interval (CI) 1.5–4.7%). The prevalence was non-significantly higher among villagers than pastoralists (3.6% vs. 2.0%). Assuming illness duration was 3 years and all presumptive cases started treatment, an overall crude incidence of 933 cases/100,000 was derived. Five of six presumptive cases in Djiguenni were confirmed by sputum smear microscopy, but none out of eight presumptive cases were confirmed in Néma, although the same nurse performed all clinical examinations in both departments. This result was attributed to the use of expired reagents in Néma. Communities mentioned distance rather than lack of information as the main constraint to seeking diagnosis, but poor diagnostic centre performance also delayed decision-making.

**Conclusions:**

TB prevalences were high among both pastoralists and villagers. None of the 14 presumptive cases sought prior diagnostic testing. TB diagnostic centres in the remote rural study zone were poorly equipped. These centres must remain in operation to reduce TB incidence in vulnerable communities in insecure remote rural zones and to reach national health goals.

## Introduction

The epidemiological profiles of resource-poor countries such as Mauritania are still dominated by infectious diseases, including malaria, tuberculosis (TB), and HIV/AIDS, in addition to nutritional imbalances and prenatal deaths. In Mauritania, the situation for TB remains alarming. In 2012, Mauritania was among the countries with highest TB incidences (>300 new cases/100,000) (1), although it had a comparably low HIV incidence. According to the WHO database, the incidences have decreased since 2013. The National Control Program against Tuberculosis and Leprosy in Mauritania (PNLTL) reported slightly lower numbers than the WHO estimates: in 2013, an annual incidence of 115/100,000 and a prevalence of 203/100,000 (2). These incidence estimates are three times lower than those of 2007. Case detection was estimated by the WHO, as in 2013, at around 50% (3). None of these estimates were derived from a population-based study. A PubMed search (www.ncbi.nlm.nih.gov/pubmed) revealed nine publications on TB in Mauritania in the last 25 years. These publications included reports on TB in livestock (two on pulmonary TB in camels) and specialised human case studies (for instance spondylodiscitis), but none were population-based surveys.

TB remains one of the top priority health issues in Mauritania. The first Global Fund to Fight AIDS, Tuberculosis and Malaria (the Global Fund) grant was received for TB in Round 2 (2006). However, Global Fund funding was not sustained for managerial reasons. The HIV prevalence among TB patients increased dramatically, although it was still at a rather low level, climbing from 0.5% in 1986 to 1.4% in 1996 and 5.2% in 2003 (4). Geographical and way of life factors, such as mobility in remote extensive livestock production systems, as well as economic, social, cultural, and political factors, likely influence the success of detection and treatment in Mauritania. In 2006, treatment success was 41%, much lower than the African average of 76%. According to the last census in 2000 when mobile pastoralists (‘nomads’) were separately counted, they represented 5% of the total population (ONS (5)), which was lower than the counts of 12% in 1988 and 70% in 1965. However, the 2000 census was carried out during a different season, when large numbers of transhumant pastoralists could potentially have crossed borders to use ‘dry season pastures’, for example in Mali. The most recent census, in 2013, did not record mobile pastoralists as differentiated from settled communities. Although rural zones continue to support important pastoralist populations, it was argued that urbanisation has progressed in Mauritania.

The relationship between migration, population mobility, and health received new attention as the Millennium Development Goals came into focus. Gele et al. (6) found one of the highest reported TB patient delays to seek diagnosis and adequate treatment among Ethiopian pastoralists. This delay was associated with inadequate pastoralist knowledge of TB and distance to the nearest healthcare facility (6). Mobile pastoralists in arid and semi-arid regions with an annual grass cycle and where crop farming is not feasible continuously move seeking sufficient pasture for their livestock. Their mobility, together with marginalisation from primary education when compared to settled communities (7), restricts their access to information, which is further jeopardised as pastoralists are rarely represented on the local health committees that inform communities on health issues. Sociology studies in south-eastern Mauritania show that pastoralists perceive TB as incurable and hereditary (8, 9). The aim was to improve healthcare for TB patients in south-eastern Mauritania. We planned an epidemiological study to determine if, as previously reported but poorly documented, TB incidences were higher among pastoralists than in settled populations.

## Methods

### Study sites and population and field team

**Fig. 1:**
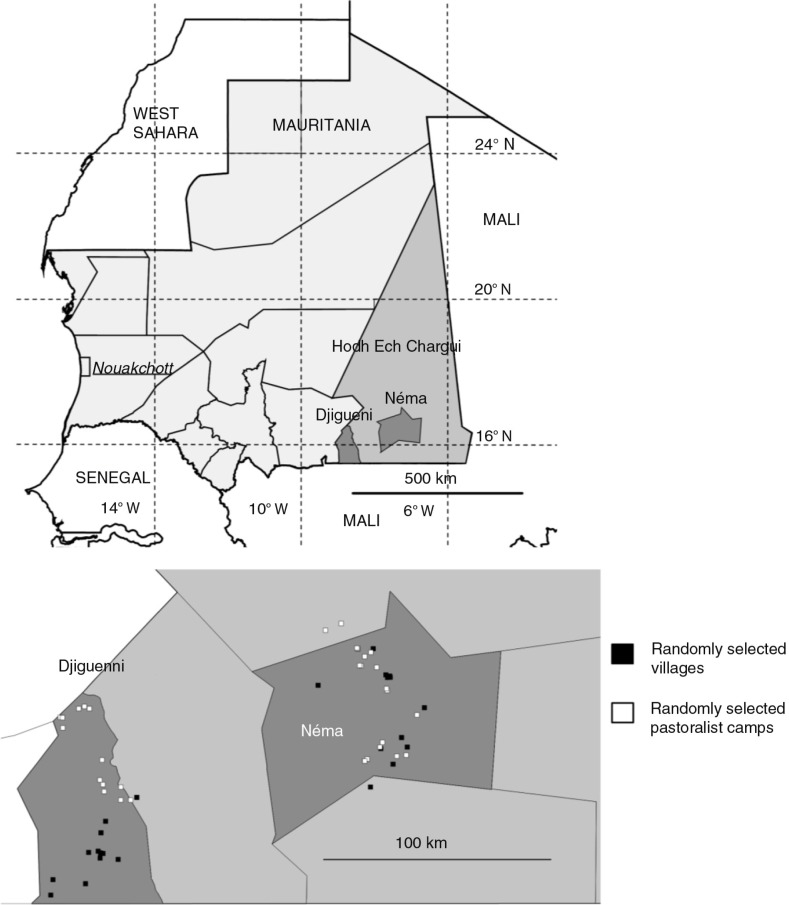
Map of Mauritania showing the two departments (Djiguenni and Néma) of Hodh Ech Chargui Province where the study was conducted. The locations of the randomly selected villages and pastoralist camps are shown with black and white squares, respectively.

We used a combination of quantitative and qualitative methods to account for population-based estimates and risk factors of TB infection as well as to capture the perspective of service providers and communities. The study sites were in the *wilaya* (‘region’) of Hodh Ech Chargui in south-eastern Mauritania (Fig. 1). Hodh Ech Chargui has the highest livestock numbers in Mauritania, given its vast pastures. The population of Hodh Ech Chargui was 430,668 in 2013 (10). Transhumance of pastoralists traditionally occurred over 7–8 months in Mali and 4–5 months in Mauritania. However, during our study there were very restricted movements in Mali due to conflict situations with Al-Qaeda involvement in northern Mali.

There are 30 municipalities of Hodh Ech Chargui administered in six departments (*moughataa*). Because of the vast size of Hodh Ech Chargui and current security concerns, we purposively selected the departments of Djiguenni and Néma as study sites based on a prior social science study on TB illness among pastoralists (9).

The field team included a driver, a guide, a nurse from the Néma TB diagnostic centre, and an epidemiologist. Participants were enrolled in the field study between 2 January and 10 February 2012. We interviewed 13 health staff (medical officers, nurses, and laboratory technicians) in Néma and Djiguenni in order to select the person with the best clinical experience for the position of study nurse. The approximate numbers of inhabitants of the seven municipalities in Djiguenni and 10 municipalities in Néma were known.

### Sample size

The cluster sample size calculation for clinical TB cases (presumptive TB cases) was calculated with the formula of Bennet et al. (11) and took an intraclass correlation coefficient of 0.2 and an expected clinical prevalence of 5% among adults. The high assumed prevalence was derived from personal communications on untreated TB prevalence in the region (regional coordinator of the national TB programme, Mohamed Mahmoud Ould Taleb, personal communication December 2010) and a study among mobile pastoralists of Chad, which reported a prevalence of 4.6% (12, 13). We conducted a comparative study between pastoralists and the settled communities, whereby the sample size calculation was to obtain prevalence estimates with sufficient precision for both pastoralists and villagers. A sample size of 10 people from 22 villages/pastoralist camps (i.e. 220 participants) would achieve, with a design effect of 2.8, a precision not wider than 5 percentage points, corresponding to a 95% confidence interval (CI).

### Selection of participants and ethical statement

We used a random selection proportional to size to select three municipalities in Djiguenni and four in Néma. Within the seven selected municipalities, all villages and city areas (of the cities Djiguenni and Néma) were listed (17 and 13, respectively), and 11 were selected proportional to size in Djiguenni while all were retained in Néma. Within a selected village or city area, a pen was spun at the centre point (mosque, water point, or market). Using the indicated direction and moving away from the centre point, all household names were listed with the help of the elders and papers with the names of the households were put in a bag. A household was defined as people living under the same roof and sharing meals together. Ten households was drawn from the bag that contained all names. The names of the pastoralist camps were compiled with knowledgeable people such as health staff, representatives of pastoralists, and village elders in the selected villages. A total of 25 camps in Néma and 21 in Djiguenni were listed, of which a simple random selection was done with random numbers generated using Microsoft^®^ Excel. Households (tents) in a camp were selected by spinning a pen at its centre.

Within a selected household/tent, the purpose and procedure of the study were explained to all members present. The inclusion criteria were men and women who were at least 15 years old. Names of household members present and willing to participate were put into a bag and one name drawn randomly. Informed written consent was obtained from all participants enrolled. With regard to ethics statement, we obtained a research permit (no. 385/11 December 2011) from the Ministry of Health. All interviews and examinations were done in a private place. TB presumptive cases were referred to the nearest TB diagnostic centre and all received TB treatment (with financial support for transportation when needed).

### Clinical examination, microscopy, and questionnaires

The clinical examinations and classification as a presumptive TB case were done by a nurse specifically trained for TB clinical diagnosis by the PNLTL, who used their protocol (2) and had several years of experience. A presumptive (pulmonary) TB case had a persistent productive cough, possibly with haemoptysis, and/or pathologic pulmonary auscultation, prolonged fatigue, night sweats, or weight loss. Presumptive cases were referred to a technician trained in microscopy by the Mauritanian National Reference Laboratory for Mycobacteria in collaboration with the national programme. All presumptive cases were further referred to the TB diagnostic centre in their department, where they were re-examined and asked to submit two to three early morning sputum smears, which were stained with Ziehl-Neelsen stain for microscopic detection of acid-fast bacilli.

The questionnaires were in French, but the questions were asked of participants in Hassaniya (the variety of Arabic spoken in Mauritania). The questionnaire included 1) socio-demographics, 2) sources of income and transhumance routes, 3) access to and use of healthcare, 4) knowledge of TB, and 5) knowledge of HIV/AIDS and TB co-infection. In addition, two focus group discussions (FGDs) were conducted in communities with an interview guide on perception of different health services and prevailing diseases.

### Data management and analyses

Questionnaire data were double entered in Microsoft^®^ Access and compared and corrected with the Epi Info 3.5.1 module ‘data compare’. We adjusted the univariate models to way of life (villager or pastoralist), sex, and age class (15–45 or ≥45 years) to also present adjusted odds ratios (ORs) with their 95% CIs. To consider possible clustering of TB suspects within villages or camps in the calculation of prevalence, we used the command xtgee with a random effect (re) at the village or camp level in Stata^®^ IC 12 (StataCorp LP, College Station, TX, USA). Because clustering was minimal, that is, the random effect model did not increase the CIs, we report here the binary exact CIs for prevalences. We considered a level of significance at a *p*-value of ≤0.05. The minimal distances of participants to a health post, health centre, and the regional hospital were calculated with the geodist command using the coordinates of the nearest health structure and of the villages or camps. The distance was then analysed with the Wilcoxon rank-sum test.

Data from the qualitative survey were transcribed into French from audio recordings and summarised with the use of MAXQDA software.

### Conversion of prevalence to a crude incidence

To determine the crude incidence of presumptive TB cases in the population, the proportion of found presumptive cases was divided by the duration of disease, which was assumed to be 3 years. Tiemersma et al. found that the duration of TB from onset to cure or death was approximately 3 years and seemed similar for smear-positive and smear-negative TB (14). Using the assumption that the duration is related to prevalence and incidence, with the WHO and PNLTL estimates for Mauritania, the calculation of duration=incidence/prevalence gave 1.7 and 1.5 years for the duration of TB, respectively. However, we have retained the more conservative estimate of 3 years of untreated TB, because shorter durations (i.e. 2 years) likely underestimate the duration of disease (14).

## Results

Our sample was composed of 250 villagers and 250 pastoralists, and we had complete examinations of all participants and questionnaire data sets for 486. Ten participants per site were enrolled in 11 villages and 12 camps in Djiguenni and 10 villages and 13 camps in Néma. In two large villages of Djiguenni and Néma, 20 and 40 participants were exceptionally enrolled, respectively (Fig. 1). One participant was of the Pullar ethnic group and all others were Maure. The median household size was six in villages and seven in pastoralist camps. In our sample, we had more women than men (61% vs. 39%), but the percentage of women was slightly lower among villagers than pastoralists, at 58 and 64%, respectively. The higher proportion of women in the sample likely occurred because we included people who were present at the time the study team arrived and women are more likely to be at home during the day. There were no significant differences in age classes between the villagers and pastoralists. The minimal distances between the villages and a health post, health centre, and regional hospital were 17.9 km (12.6–35.8), 18.5 km (2.2–39.8), and 67.1 km (37.6–190.9), respectively. The distances between pastoralist camps and these structures were 15.9 km (7.8–24.4), 43.4 km (35.4–52.7) and 52.4 km (40.6–167.8). The distances did not differ significantly for villagers and pastoralists.

Fourteen presumptive TB cases were recorded among the 500 examined, none of whom were diagnosed at a TB centre or suspected to have TB by a nurse prior to this study. We found eight presumptive cases in Néma and six in Djiguenni in a total of 12 villages/camps. The overall prevalence of suspects and the prevalence calculated with the random effect model were 2.8 (95% CI 1.5–4.7) and 2.8 (1.6–4.7). The prevalence was non-significantly higher among villagers, at 3.6% (1.7–6.7) compared to the 2.0% (0.7–4.6) among pastoralists. Our prevalence of new presumptive TB cases translated to an overall crude incidence of 933 cases/100,000 (95% CI 500–1,567). Regarding the risk factors for being a presumptive TB case, age ≥45 years was significantly associated, although not in the adjusted model (Table 1). As expected and serving as confirmation on how presumptive cases were classified, having at least one cardinal symptom, pathologic pulmonary auscultation, cough, and fever were associated highly significantly (*p*<0.01) with being a presumptive TB case in both univariate and adjusted models.

**Table 1:** Association of risk factors of presumptive tuberculosis cases in Hodh Ech Chargui, Mauritania

		Presumptive tuberculosis cases					
				
		No		Yes			OR adjusted to way of life, sex, and age class
				
		*n*	%	*n*	%	Crude OR (95% CI)	
Way of life	Villager	241	96.4	9	3.6	1	1
	Pastoralist	245	98.0	5	2.0	0.5 (0.2–1.7)	0.6 (0.2–1.8)
Age class	15–45	310	98.7	4	1.3	1	1
	≥45	176	94.6	10	5.4	3.5 (1.1–11.3)*	3.1 (0.9–10.4)
Sex	Female	298	98.0	6	2.0	1	1
	Male	188	95.9	8	4.1	0.5 (0.2–1.4)	0.7 (0.2–2.1)
Marital status	Single	88	98.9	1	1.1	1	1
	Married (monogamous)	305	97.1	9	2.9	2.6 (0.3–20.8)	1.4 (0.1–13)
	Married (polygamous)	1	100.0	–	–	–	–
	Divorced	44	97.8	1	2.2	2 (0.1–32.7)	2.4 (0.1–41.4)
	Widowed	48	94.4	3	5.9	5.5 (0.6–54.3)	2.8 (0.2–38.3)
Distance to health centre	≤37.5 km	240	96.0	10	4.0	1	1
	>37.5 km	246	98.4	4	1.6	0.4 (0.1–1.2)	0.4 (0.1–1.4)

OR, odds ratio; CI, confidence interval.

* *p*-value≤0.05,

** *p*≤0.001, and

*** *p*≤0.001.

All 14 presumptive TB cases were referred to the TB centre of their department for sputum smear microscopy for acid-fast bacilli. At the TB centre in Néma, all eight suspected cases were determined to be smear-negative pulmonary TB, whereas in Djiguenni, five of the six presumptive cases were confirmed to be smear-positive pulmonary TB (Table 2). In Néma, the interviewed technicians mentioned repeatedly during interviews that they were using expired reagents. Based on these statements and the fact that the same nurse made the diagnosis of all presumptive TB cases, we concluded that a similar proportion of smear positives (i.e. 80% of tested presumptive cases) would likely have been found in Néma if the reagents were not expired. During the exploratory visit prior to the study, the technicians in Néma did not specifically mention that they periodically only had access to expired reagents, but they stated that working conditions were inadequate for reasons such as only one working microscope across the entire department. All 14 presumptive TB participants started treatment based on their clinical symptoms. In the remote diagnostic centres, treatment was also initiated for patients presenting with cardinal TB symptoms but inconclusive laboratory results, because technicians were aware of the laboratory constraints, particularly expired reagents.

**Table 2:** Detection of new presumptive and smear-positive pulmonary TB cases among villagers and pastoralists in the departments of Djiguenni and Néma of Hodh Ech Chargui, Mauritania

	Villages		Pastoralists	
		
Department	Smear positive	Smear negative	Smear positive	Smear negative
Djiguenni	2	1	3	0
Néma	0	6	0	2
Total	2	7	3	2

*Note*: The presumptive TB classification was done by the same TB nurse and same protocol. TB, tuberculosis.

### TB awareness in the communities

The general population had hardly any access to information and TB awareness was low. In the past, NGOs have been commissioned to raise awareness and disseminate information, but these campaigns were for a limited time and not continuous. The financial aspects of coping costs for seeking diagnosis, such as long travel time and absence from work, were the most commonly mentioned obstacles for visiting a TB diagnostic centre. Additionally, disrespectful treatment from health workers, the perceived low quality of service, and lack of information and awareness were frequently cited. In the FGDs, the following diseases were consistently ordered from most to least important: malaria, acute respiratory illness, diarrhoea, skin diseases, rheumatism, gastroenteritis, and anaemia. The local names for *tuberculosis* were *kouha twila* (‘persistent cough’), *kouha* (‘cough’) *seehle*, *twile* (‘persistent’), *gachouche*, *kouha msarine*, *kouha ataama* (‘bone cough’). The various names and the possibility of stigma may be reasons why none of the local terms for TB illness were listed among the most important diseases. Most of the people in the area consult local healers. According to interviews with health staff, only the health centre of Néma informed patients about possible co-infections of TB and HIV/AIDS, and sometimes patients were referred for further examination to the capital Nouakchott, which is nearly 1,000 km away.

## Discussion

This work reports the first population-based TB prevalence study in Mauritania. An overall prevalence of (new) presumptive TB cases among adults in a remote rural zone of Mauritania of 2.8% (translating into a crude incidence of 933 cases/100,000) is notable – and it is high among both villagers and pastoralists, as well as being nine times higher than that reported for the whole country in national reports. A recent multisite study found that case detection interventions increased sputum smear-positive detection by 36.9% (15). Potential extrapulmonary cases were not further considered, despite the fact that extrapulmonary TB cases represent a setting-specific 5–20% of all TB cases (16). Sahelian cattle can be infected with *Mycobacterium bovis*, which can cause both pulmonary and extrapulmonary TB in people (17). However, the zoonotic potential of *M. bovis* in sub-Saharan Africa is currently known to be less substantial than initially expected at the height of the HIV pandemic 20 years ago (18). A recent review found median proportions of bovine TB of 2.8% among human TB patients in Africa and 1.4% in the rest of the world (19).

Laboratory diagnostic capacity was likely heavily influenced by the use of expired reagents, lack of supplies in laboratories, and only one functional microscope at the health centre that served the entire department of Néma, where there was also no working refrigerator. Interviewed technicians reported that periods between quality controls were often longer than the planned 3-year intervals. After a patient is diagnosed with TB, a follow-up should take place in the second, fifth, and eighth months. However, loss to follow-up is not recorded in these remote communities. Logistical constraints are the reality in remote TB centres. However, well-trained and committed health workers continue to provide care in these zones and should be better supported by national programmes.

We used all presumptive TB cases to calculate the overall crude incidence because none of the presumptive cases previously sought diagnosis (thus all were new to the registration system). This decision was also based on the fact that five of the six identified clinical presumptive cases were confirmed by microscopy in the department of Djiguenni, where there was an operational microscope, giving a higher proportion than the usual 45–60% of pulmonary presumptive cases confirmed by microscopy. Although laboratory confirmation was extrapolated from one confirmation centre to the other, which constitutes a limit of this study, we believe it is justified because presumptive cases were established in the same way in both departments.

We did not confirm the hypothesis that pastoralists had higher TB incidence than villagers. It was more difficult than anticipated to differentiate the lifestyle between settled agro-pastoralists and transhumant and mobile pastoralists. Many pastoralists have settled in recent years and villagers and pastoralists are often closely related. The reasons for increasing settlement are better scolarisation of children and security concerns. In the advanced stages of TB illness, interviewees stated that long, difficult journeys could only be made with an accompanying person. For livestock owners, a long absence from the herd meant that livestock had to be entrusted to somebody else. The movements of the campsites could be challenging because it often made it impossible for patients to visit a health centre repeatedly to maintain long-term treatment, as required for TB (20). In the centres, communication with staff was often poor because there were many different languages spoken (21). The fact that we did not find higher incidences among pastoralists than villagers in Hodh Ech Chargui indicates that the barriers to accessing TB diagnosis are comparable. The average distances for both groups were half a day's walk to the closest health centre and a full day by horse-drawn cart to the nearest hospital.

## Conclusions

We found a high prevalence of presumptive TB cases among both villagers and pastoralists in this population-based study in remote rural Mauritania. The newly identified presumptive TB patients would have been undiagnosed and untreated in the absence of this study. The TB diagnostic centres of the national TB programme in rural south-eastern Mauritania were poorly equipped in the rural study zone. One of the two centres was not operational, due to expired reagents, despite the presence of well-trained and committed technicians and nurses. The communities stated distance as the main constraint to seeking diagnosis at a facility rather than a lack of information. The weak performance of the diagnostic centres also influenced their decision on whether to seek diagnosis, and from previous social science studies we know that adapted information campaigns increases TB awareness in pastoralist communities, who more often than other communities cannot access good information. The TB centres in the sometimes-insecure study zone, where health staff nonetheless continue working, must be kept operational in order to not exclude potentially vulnerable populations and reach national health goals. This aim seems achievable with sufficient financial means and firm commitment ranging from international to local levels.
